# Understanding the Role of Transition Metal Oxides as Hole-Selective Contacts for Enhanced Efficiency in Selenium Solar Cells

**DOI:** 10.1021/acsaem.5c02637

**Published:** 2025-11-06

**Authors:** Oriol Segura-Blanch, Arnau Torrens, Ivan Caño Prades, Alex Jimenez-Arguijo, Laura Garcia-Carreras, Lorenzo Calvo-Barrio, José Miguel Asensi, Joaquim Puigdollers, Marcel Placidi, Edgardo Saucedo

**Affiliations:** † 16767 Universitat Politècnica de Catalunya (UPC), Photovoltaic Lab − Micro and Nano Technologies Group (MNT), Electronic Engineering Department, EEBE, Av Eduard Maristany 10-14, Barcelona 08019, Catalonia, Spain; ‡ Universitat Politècnica de Catalunya (UPC), Barcelona Center in Multiscale Science & Engineering, Av Eduard Maristany 10-14, Barcelona 08019, Catalonia, Spain; § Centres Científics i Tecnolígics(CCiTUB), 16724 Universitat de Barcelona, Carrer de Lluís Solé i Sabarís 1, 08028 Barcelona, Spain; ∥ IN2UB, Departament d′Enginyeria Electrínica i Biomídica, 216515 Universitat de Barcelona, Carrer de Martí i Franquès 1, 08028 Barcelona, Spain; ⊥ IN2UB, Departament de Física Aplicada Universitat de Barcelona, Carrer de Martí i Franquès 1, 08028 Barcelona, Spain; # Institut de Recerca en Energia de Catalunya (IREC), Jardins de les Dones de Negre 1, Sant Adrià del Besòs 08930, Catalonia, Spain

**Keywords:** photovoltaics, wide band gap, selenium, hole transport layer, transition metal oxides, indoor PV

## Abstract

Selenium solar cells (SeSCs) are gaining renewed interest as wide band gap photovoltaic absorbers suitable for indoor energy harvesting and tandem applications. While significant progress has been made through extensive optimization of electron transport layers (ETLs), the role of hole transport layers (HTLs) has been comparatively less explored. In this work, we investigate the integration of inorganic transition metal oxides (TMOs), namely molybdenum oxide (MoO_*x*_), tungsten oxide (WO_*x*_), and vanadium oxide (V_2_O_*x*_), as hole-selective contacts in SeSCs. We systematically optimize the TMO thicknesses and assess their effect on device performance under both standard AM1.5G and indoor illumination conditions. Our results demonstrate that incorporating optimized TMO layers substantially improves the fill factor (FF) and parasitic resistances of the device, leading to enhanced power conversion efficiencies (PCEs). The best outdoor performance is achieved with a 20 nm MoO_*x*_ HTL, delivering a champion PCE of 5.5%. For indoor conditions, a 10 nm V_2_O_*x*_ HTL enables PCE values exceeding 10% across a wide range of light intensities and spectra. Ultraviolet photoelectron spectroscopy and transmission electron microscopy-energy dispersive X-ray spectroscopy analyses reveal strong interfacial interactions between Se and the TMOs, including evidence of spontaneous MoSe_2_ formation at room temperature, which likely contributes to enhanced hole selectivity and suppressed recombination. Additionally, preliminary indications suggest the possible formation of VSe_2_ under similar conditions. These findings underscore the crucial role of inorganic HTLs in unlocking the full potential of SeSCs and highlight their suitability for emerging applications such as indoor photovoltaics and monolithic tandem architectures.

## Graphical abstract

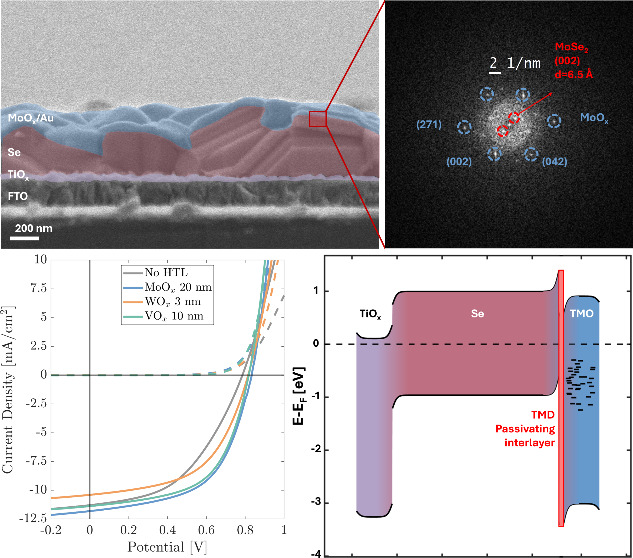

## Introduction

Selenium had a pivotal role in the discovery of the photoconductivity^1^ and photovoltaic (PV) effects,^2^ as well as being the absorber layer of the first solid state solar cell.^3^ However, since those early developments, Se has been largely overshadowed by the success of silicon, the dominant absorber material in commercial PV devices.^4^ In recent years, selenium has gained renewed interest as an attractive photovoltaic absorber. The resurgence is driven by its wide band gap of 1.8–2 eV^5^ and low processing temperatures (melting temperature of ∼217 °C^6^), which make it a promising candidate for both established and niche photovoltaic applications where crystalline silicon is not suitable. Currently, silicon solar cells have achieved power conversion efficiencies of up to 27.8%,^7^ approaching the theoretical radiative limit for single-junction devices.^8,9^ Moreover, tandem photovoltaic devices can reach even higher efficiencies, as they are composed of a wide band gap top cell and a narrow band gap bottom cell, allowing more effective utilization of the solar spectrum by reducing thermalization. Nevertheless, these devices require gentle processing conditions to prevent damage to the bottom cell, a challenge that selenium addresses, as demonstrated by the first selenium–silicon tandem devices with PCEs ranging from 2.7%^10^ for monolithic architectures to the 5.7%^11^ for bonded single junction cells. These initial demonstrations establish a strong foundation for future improvements in selenium-based tandem architectures.

Indoor photovoltaics (IPV) is a promising approach for in situ energy harvesting to power small electronic devices using ambient light within buildings. With the rise of the Internet of Things (IoT), which relies on extensive sensor networks to collect large amounts of data, IPV is becoming increasingly relevant. Sensors in IoT networks are typically powered by batteries, which can lead to significant maintenance challenges including battery replacement and waste management. Pairing IoT sensors with photovoltaic devices capable of recharging their batteries from indoor illumination offers a potential solution for efficient deployment.^12^ However, conventional crystalline silicon cells are unsuitable for indoor light power conversion due to their low band gap.^13^ Selenium-based solar cells are a promising alternative, as their band gap is well-matched to the emission spectra of commonly used LED lighting.^14–16^ SC improvement has been based on the electron transport layer (ETL) optimization. First, Nakada et al.^17^ reached 5% power conversion efficiency thanks to replacing the previously used ZnO and SnO_2_ ETLs with a sputtered titanium dioxide (TiO_2_) film. Later, Todorov et al.^18^ in 2017 broke the 6% PCE barrier with an ultrathin selenium absorber of just 100 nm more than 30 years after the last milestone in this technology. They claimed that this performance increase was due to refinement of the ETL using a sputtered layer of zinc magnesium oxide (ZnMgO) with the optimal Zn to Mg ratio. That resulted in an enhancement of the fill factor and open circuit voltage (*V*_OC_). This breakthrough sparked renewed interest in selenium-based photovoltaics. Remarkably, in a short period of time, the Ding-Jiang Xue group presented two new world records: Wenbo Lu et al. with a 7.2%^19^ efficiency in March 2024 and Qingxiang Liu et al. with an 8.1%^20^ efficiency in November 2024 under AM1.5 illumination, as well as reporting outstanding indoor performances of 18%^19^ and 20.1%^21^ under 1000 lx illumination. All of these recent performance achievements are based on a TiO_2_ ETL synthesized by spray pyrolysis and 1 μm thick selenium absorber. However, they attribute their groundbreaking results to the controlled orientation of the selenium ribbons in the selenium trigonal structure by tuning the crystallization and substrate deposition temperatures.^22^

Even though extremely efficient, the SeSC of Todorov is the only record device that uses a hole transport layer, with the rest relying solely on a gold back contact. Todorov uses a 15 nm thick thermally evaporated molybdenum oxide HTL, reporting that this layer increases the FF by more than 10% and the *V*_OC_ by more than 100 mV. On the one hand, evaporated or sputtered MoO_*x*_ is the only inorganic HTL reported for SeSCs.^10,23–25^ Indeed, molybdenum oxide is known to work as a hole selective film in almost any of the existing photovoltaic technologies.^26–34^ On the other hand, several organic molecules and polymers have been tested as HTLs for Se devices, including PTAA,^35^ (DPP)-based polymers,^36^ P3HT,^37^ and PEDOT:PSS.^38^

In this work, we explore the integration of transition metal oxides, namely, molybdenum oxide (MoO_*x*_), tungsten oxide (WO_*x*_), and vanadium oxide (V_2_O_*x*_), as HTLs in SeSCs. These three oxides were selected due to their high work functions, proven compatibility as hole-selective contacts in other photovoltaic technologies, and their thermal and chemical stability under the mild processing conditions required for SeSCs. By systematically evaluating the impact of these transition metal oxides (TMOs) on device performance, we identify their role in enhancing power conversion efficiency, particularly under low light conditions relevant for indoor photovoltaics. Our results show that carefully optimized TMO layers can increase the fill factor and open-circuit voltage by significantly improving the shunt resistance while minimizing series resistance, achieving a champion cell under AM1.5G using 20 nm of MoO_*x*_. Notably, the introduction of a 10 nm thick vanadium oxide layer led to the best indoor PCE values across a broad range of illumination conditions, with molybdenum oxide also delivering excellent performance. Transmission electron microscopy-energy dispersive X-ray spectroscopy (TEM-EDXS) and ultraviolet photoelectron spectroscopy (UPS) analyses reveal substantial interfacial interactions between Se and the TMOs, including evidence of spontaneous MoSe_2_ and VSe_2_ formation at room temperature. These interfacial features appear to play a critical role in the improved charge extraction and suppression of nonradiative recombination. This study provides new insights into the physics of the Se/TMO interface and demonstrates the potential of inorganic HTLs to unlock high-efficiency selenium photovoltaics for both outdoor and indoor applications.

## Experimental Section

### Solar Cell Fabrication

FTO-coated glass substrates (Sigma-Aldrich, TEC 15, ∼13 Ω/sq) were purchased from Sigma-Aldrich and ultrasonically cleaned in acetone, isopropanol, and Milli-Q water and dried using a nitrogen gun. A compact TiO_2_ layer was then deposited onto FTO glass via spray pyrolysis at 500 °C from a mixed solution of titanium diisopropoxide bis­(acetylacetonate) (Sigma-Aldrich, 75 wt % in isopropanol) and ethanol (96%) in the ratio of 1:19 by volume followed by a final annealing at 500 °C during 20 min. The absorber layer was deposited using a Kenosistec coevaporation system at a base pressure lower than 5 × 10^–7^ mbar. First an ultrathin ∼1 nm layer of tellurium (Sigma-Aldrich, pellets, 99.999%) seed layer was evaporated, immediately followed by the deposition of 300 nm of selenium (Thermo-Scientific, 200 mesh, 99.999%) at an evaporation rate of 10–14 nm/min, without any substrate heating.

Molybdenum oxide (Sigma-Aldrich, powder, 99.97%), tungsten oxide (Sigma-Aldrich, powder, 99.995%), and vanadium oxide (Sigma-Aldrich, powder, 99.95%) thin films were deposited using a thermal evaporator system located inside a MBraun 200B glovebox under a nitrogen atmosphere. The deposition vacuum was lower than 10^–5^ mbar and at a rate of 0.3–0.6 Å/s monitored with a calibrated quartz crystal microbalance. Gold back contacts (Neyco, 99.99%) were deposited in the same system at a rate of 1–2 Å/s using a shadow mask, resulting in a solar cell active area of 7 mm^2^.

The selenium layer was crystallized on a hot plate in air at 200 °C for 5 min under two different conditions: (i) immediately after the evaporation of Te/Se, as this is the most commonly used method in the literature, or (ii) after Au evaporation, since crystallizing selenium with capping layers has been reported to improve the HTL/Se interface.^25^ For clarity, we will hereafter refer to process (i) as precrystallized and process (ii) as postcrystallized. The crystallinity of the obtained selenium absorber was examined by X-ray diffraction (Figure S1), which clearly shows that the initially amorphous Se layer crystallizes into the desired trigonal phase for photovoltaic applications. The resulting solar cells exhibited the architecture shown in Figure 1a. The absorber layer consisted of columnar grains with heights comparable to the total absorber thickness, as observed in Figure 1b,c. In all fabricated batches, a reference device without an HTL was included to decouple the effect of the hole transport layer from variations in the selenium absorber quality and the TiO_*x*_ electron transport layer, which can arise from batch-to-batch differences over the course of the study.

**Figure 1:**
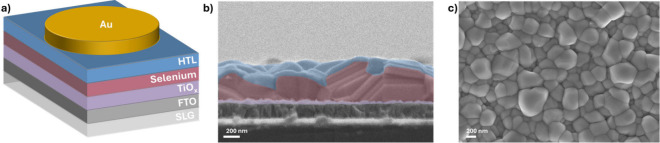
(a) Schematic illustration of the device architecture. (b) Cross-sectional SEM image of a complete device incorporating a MoO_*x*_ hole transport layer. (c) Top-view SEM image of the selenium absorber layer.

### Characterization

The current–voltage (I–V) characteristics were measured using a four-probe Keithley 6430 source meter. The I–V curves under illumination were measured using a calibrated AAA solar simulator (G2 V) LED-based with a light intensity of 87.8 mW/cm^2^ (from 300 to 1800 nm) equivalent to the standard 100 mW/cm^2^ in the full spectrum of AM1.5G conditions. To obtain the indoor performance of the studied devices, we tuned the solar simulator to output five different color temperature light spectra (2700, 3000, 4000, 5000, and 6000 K) and four different irradiances per color temperature (from 3 to 0.01 mW/cm^2^), typically encountered in real-world indoor environments.^39–42^ This resulted in 20 different irradiation conditions covering the most common interior light conditions. Power calibration of the indoor illumination was performed by using an S120VC calibrated photodiode and a PM100D console from Thorlabs. The spectral response was measured by using an Enlitech system calibrated with Si and Ge photodiodes to obtain the external quantum efficiency (EQE).

The scanning electron micrographs (SEM) were obtained using a Neon40 Crossbeam workstation from Carl Zeiss, equipped with a GEMINI field emission electron column (4 pA–20 nA, 0.1–30 kV, 1.1 nm resolution at 20 kV) and a CANION 31 gallium focused ion beam (FIB) column (1 pA–20 nA, 5–30 kV, 7 nm resolution). The images were acquired at an acceleration voltage of 5 kV with a working distance ranging from 3 mm to 7 mm. Lamellae for transmission electron microscopy were fabricated in the same chamber using the lowest available beam power at 5 kV for both milling and final polishing.

Transmission electron microscopy (TEM) experiments were performed using a JEOL ARM 200cF (NEOARM), operated at a 200 kV accelerating voltage at CCiTUB. The microscope is equipped with a cold FEG electron gun and an aberration corrector in the condenser system. STEM-EDXS data were acquired using dual JEOL DrySD windowless detectors.

Ultraviolet photoelectron spectroscopy (UPS) experiments were performed in ESFOSCAN at CCiTUB, equipment based on the PHI VersaProbe 4 instrument from Physical Electronics (ULVAC-PHI). The measurements were done using a helium source (He I line of 21.22 eV) calibrated using a Ag sample in which the work function (WF) calculated was 4.27 eV. The analyzed area was a spot of about 1.5 mm of diameter, with the sample placed at 90° with respect to the analyzer axis, and the selected resolution for the spectra was 1.3 eV of pass energy and 0.01 eV/step. Measurements were performed with and without a bias of about 10 eV, in order to create a well-defined onset of secondary electrons for ionization energy (IE) and work function calculation purposes. Samples were placed in an ultrahigh vacuum (UHV) chamber at a pressure between 5 × 10^–10^ and 5 × 10^–9^ Torr. Moreover, in order to measure beyond the surface avoiding surface contamination (adventitious carbon mainly) sputtering with a monatomic Ar^+^ ion gun (6 mm × 6 mm and 0.5 or 4 keV) was done. This sputtering conditions were previously optimized using X-ray photoelectron spectroscopy (XPS) to ensure no chemical modification of the exposed surfaces. The samples for these measurements were prepared on to SLG/FTO substrates following the methods described previously.

UV–visible transmission and reflection spectra were measured with a PerkinElmer Lambda 950 UV–vis–NIR spectrometer. The samples for these measurements were prepared on quartz substrates following the methods described previously.

## Results and Discussion

For each HTL, devices with varying thicknesses were fabricated to identify the optimal configuration. Their performance was evaluated through light and dark J–V measurements, which were subsequently fitted to a single-diode model including nonidealities (Figure S3). From these analyses, the key photovoltaic parameters (*V*_OC_, *J*_SC_, FF, and PCE), as well as the apparent parasitic resistances (R_S_ and R_SH_), were extracted. Figure 2a, Figure S2, and Figure S3 show the open-circuit voltage, shunt resistance, fill factor, and power conversion efficiency values of the fabricated Se/TMO solar cells, measured under standard outdoor illumination conditions, for both the precrystallized and postcrystallized samples, respectively. For all tested HTLs, the *V*_OC_ does not vary significantly across different HTL materials and thicknesses, except for the precrystallized vanadium oxide cells, where a thicker V_2_O_*x*_ layer consistently reduces the *V*_OC_ (Figure S2a and Figure S3a). Nonetheless, an optimal HTL thickness was identified for each material. Under these optimal conditions, the enhancement in PCE (Figure 2a and Figure S3d) arises primarily from an improvement in the FF (Figure S2c and Figure S3c). Diode fitting of the J–V curves reveals that the FF improvement can be attributed to an increase in the R_SH_ value (Figure S2b and Figure S3b). However, although thicker TMO layers can further improve the shunt resistance, excessively thick HTLs result in lower efficiency due to an increase in the apparent series resistance (R_S_) (Table S1) due to their high resistance.^43^ Moreover, the optimal HTL thicknesses generally exhibit a series resistance lower than that of the No HTL reference samples. This improvement in R_SH_ is likely due to the physical separation introduced by the HTL, which helps block shunt pathways between electrodes through the absorber layer, and more importantly, due to the enhanced hole selectivity at the rear interface.

**Figure 2:**
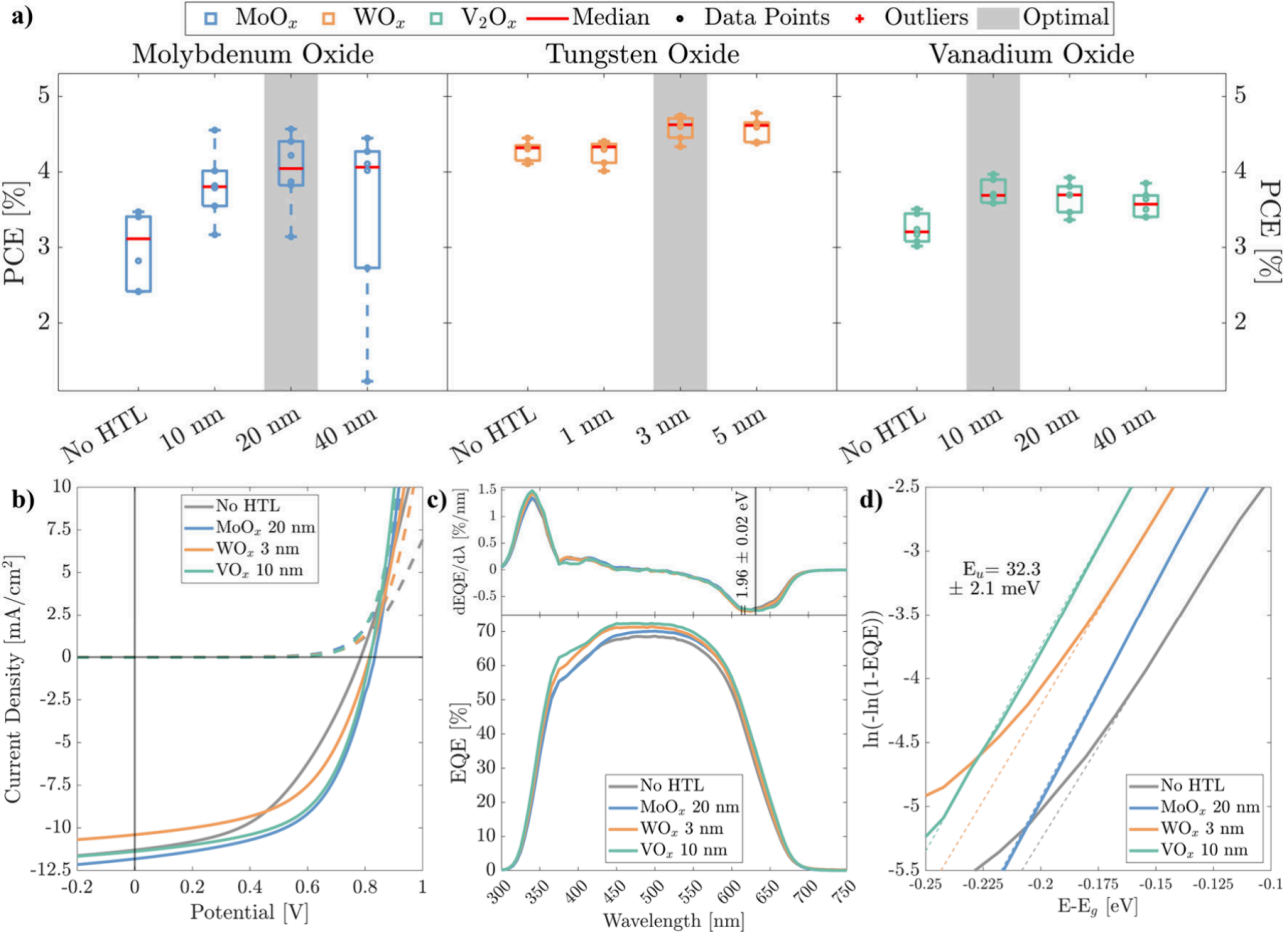
(a) Statistics over 6 representative cells of the PCE of the thickness optimization of the MoO_*x*_, WO_*x*_ and V_2_O_*x*_ hole transport layers for the precrystallized samples. (b) J-V curves of the champion cells of the precrystallized comparison cells. (c) EQE and EQE derivatives of the 4 different HTL configurations. (d) Urbach energy fitting of the previous EQEs.

Subsequently, we directly compared the performance of the optimized TMO layers, with the results summarized in Table 1 and Figure 2b. In this analysis, we focused on devices incorporating a precrystallized Se absorber layer, as both tested crystallization strategies yielded comparable performance and trends. Moreover, the precrystallization approach is the most reported method in the literature, providing a relevant basis for comparison.^17,18,20^ Notably, all tested HTLs significantly outperformed the No HTL control sample. Consistent with the trends observed during the TMO thickness optimization, the enhancement in PCE is primarily attributed to concurrent improvements in FF and *V*_OC_. Furthermore, the diode fitting analysis confirms that the increase in FF is associated with a higher R_SH_ and a reduced R_S_. The reduction in the apparent series resistance is likely linked to improved charge separation and extraction, as well as reduced recombination. While these effects may indeed lower the actual R_S_ to some extent, the more critical factor is the improved diode ideality, which can manifest as an artificial decrease in the apparent series resistance extracted from the fitting. Among the TMOs, molybdenum oxide yielded the most efficient devices, with a champion cell reaching 5.5% PCE. Vanadium oxide also delivered comparably high performance, achieving a champion cell efficiency of 5.3%. While tungsten oxide cells surpassed the No HTL reference in efficiency, their performance did not match that of the other transition metal oxides. The spectral response of these devices is shown in the external quantum efficiency (EQE) measurements (Figure 2c) and the derived optoelectronic parameters are reported in Table S2. In the 550–600 nm range, EQE values are notably higher for TMO-based samples compared to the No HTL reference, suggesting improved charge separation at the rear interface of the absorber due to enhanced depletion of the back absorber region enabled by the studied TMOs. The EQE-derived *J*_SC_ values are slightly lower than those extracted from the J–V analysis, likely due to a spectral mismatch of the solar simulator and small discrepancies between the actual and measured device area. Nevertheless, the same *J*_SC_ trends observed in the J–V curves are also reproduced in the EQE-derived values. However, the EQE-derived band gap (E_g_) and Urbach energy (E_U_) (Figure 2c,d) remain consistent across all samples, as they were fabricated from the same Te/Se batch, confirming that variations in HTLs did not affect the absorber layer optoelectronic properties.

**Table 1:** Photovoltaic Parameters of the Optimized Cells for the 3 HTL Configurations Tested and the Reference No HTL Device for the Precrystallized Samples^a^

HTL	V_OC_ [mV]	J_SC_ [mA/cm^2^]	FF [%]	PCE [%]	J_0_ [A/cm^2^]	n	R_S_ [Ω·cm^2^]	R_SH_ [kΩ·cm^2^]
No HTL	790 ± 10	10.7 ± 0.4	48.7 ± 1.7	4.1 ± 0.1	(1.6 ± 0.4) × 10^–7^	3.4 ± 0.2	37.1 ± 8.1	27 ± 10
MoO_*x*_ 20 nm	840 ± 10	11.6 ± 0.2	54.9 ± 1.3	5.3 ± 0.1	(2.2 ± 0.4) × 10^–8^	2.7 ± 0.2	22.8 ± 17.8	304 ± 125
WO_*x*_ 3 nm	810 ± 15	10.1 ± 0.2	52.8 ± 1.6	4.5 ± 0.1	(7.2 ± 0.3) × 10^–8^	3.0 ± 0.3	25.3 ± 6.8	172 ± 93
V_2_O_*x*_ 10 nm	830 ± 10	11.4 ± 0.3	55.0 ± 1.7	5.2 ± 0.1	(9.8 ± 0.3) × 10^–9^	2.5 ± 0.2	16.7 ± 4.5	183 ± 52

a The error was computed as the standard deviation (except for the error of *J*_0_ and *R*_SH_ where the standard error of the mean is used due to their logarithmic nature.

The performance of these optimized devices was evaluated under representative indoor lighting scenarios, presenting the photovoltaic parameters as a function of illumination conditions in Figure 3 and Figure S4, constructed using the data from Tables S3–S5. The most efficient device featured a 10 nm thick V_2_O_*x*_ HTL, exhibiting over 10% PCE across most of the tested illumination conditions and especially at high color temperatures, where it reached 10.58% PCE. The second-best performance was achieved by the MoO_*x*_ device, maintaining efficiencies above 8.43%, while the No HTL reference reached only about 8% PCE under the most favorable conditions. These changes in efficiency trends relative to AM1.5G illumination are intriguing and likely arise from a complex interplay of factors, including the different spectrum and intensity of indoor light, sample-to-sample variations, and/or light-induced modifications of the band alignment. Most notably, devices incorporating HTLs maintained their efficiency even under very low incident powers (<1000 μW/cm^2^), whereas the reference No HTL cell exhibited a sharp decline in performance in this regime. This resilience at low light levels stems from the enhanced *V*_OC_ and FF robustness at low injection conditions in the HTL-containing devices, which is a direct result of the increased shunt resistance enabled by the optimized hole transport layers (Figure 3d–i).^44^ This hypothesis is further supported by the fact that the *J*_SC_ maps of the three studied samples are nearly identical (Figure S5). This consistency rules out significant contributions from optical effects, such as enhanced absorption due to optical spacing, indicating that the performance differences arise primarily from the enhanced device properties due to the TMO HTLs.

**Figure 3:**
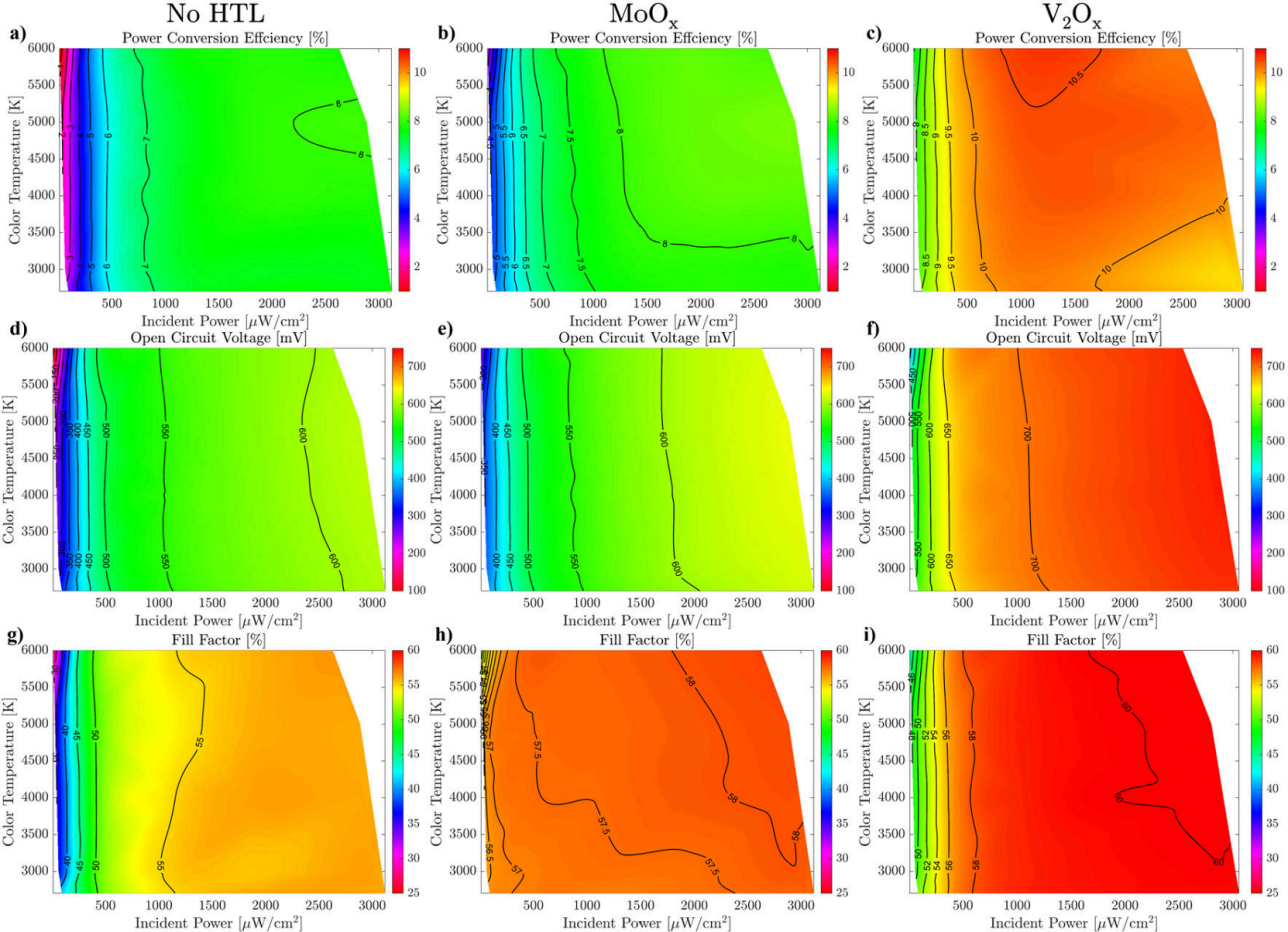
Indoor PV optoelectronic parameters maps of (a), (d), (g) No HTL sample, (b), (e), (h) 20 nm MoO_*x*_ device, and (c), (f), (i) 10 nm V_2_O_*x*_ device.

Additionally, microstructural and compositional analyses were carried out to gain further insight into the nature of the TMO/Se interfaces. To this end, we performed STEM combined with EDXS on lamellae extracted from the most efficient devices. In Figure 4, we present the STEM image and corresponding EDXS elemental maps of a lamella extracted from the champion cell featuring a 20 nm MoO_*x*_ HTL and a precrystallized selenium absorber. The STEM image clearly delineates the multilayer structure of the device, while the EDXS maps confirm the elemental composition of each layer. At the TiO_*x*_/Se interface, the elemental distribution reveals a sharp and well-defined boundary, indicating minimal interdiffusion. In contrast, the MoO_*x*_/Se interface appears more diffuse, suggesting a significant Se diffusion into the MoO_*x*_ layer. This interfacial diffusion may indicate the formation of a MoSe_2_ interlayer between the MoO_*x*_ HTL and the selenium absorber. To further investigate this phenomenon, we performed selected area electron diffraction (SAED) at the MoO_*x*_/Se interface. As shown in Figure 5, the upper region of the TEM image reveals that selenium diffused into the MoO_*x*_ layer, forming MoSe_2_. This is confirmed by the presence of a diffraction pattern corresponding to a *d*-spacing of 6.5 Å, which is characteristic of the (002) planes of the 2H-MoSe_2_ phase. Such a large interplanar distance cannot be attributed to either MoO_*x*_ or elemental selenium. The formation of transition metal dichalcogenide (TMD) interlayers at interfaces between chalcogen-rich absorbers and TMO-based transport layers has been previously reported.^45–48^ Specifically, the emergence of MoSe_2_ at the MoO_*x*_/Se interface has been observed in systems where the substrate is heated during the thermal evaporation of MoO_*x*_.^49^ Remarkably, in our study, the same phenomenon occurs at room temperature without additional thermal activation.

**Figure 4:**
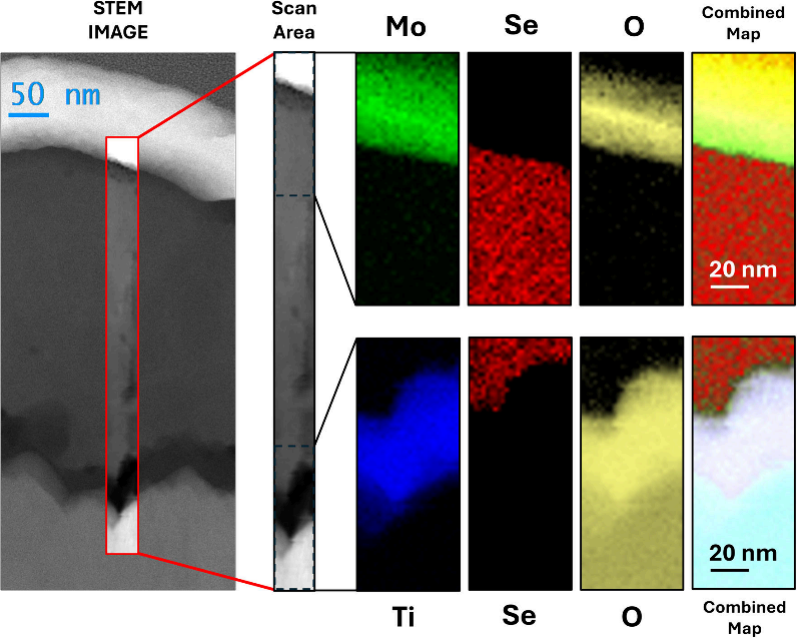
Cross-sectional STEM image and corresponding EDXS elemental maps of a lamella extracted from the champion selenium solar cell, featuring a 20 nm MoO_x_ HTL on a precrystallized selenium absorber.

**Figure 5:**
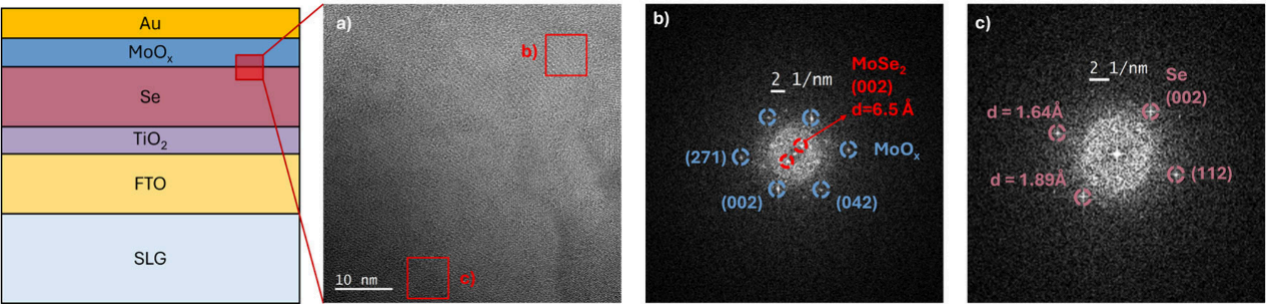
(a) HRTEM image of the Se/MoO_*x*_ interface. (b) SAED pattern of the area containing MoO_*x*_ and MoSe_2_. (c) SAED pattern of the Se area with indexed phases (ICDD 00-047-108, ICDD 01-086-2246, ICDD 03-065-3481).

Similar STEM/EDS analysis was conducted on the optimized V_2_O_*x*_-based sample (Figure S6). In this case, the interdiffusion was so extensive that the boundary between the oxide and potential selenide phase could not be clearly identified. Rather than forming a well-defined bilayer structure of TMO and TMD, a complete intermixing between vanadium oxide and selenium is suggested by the results. The formation of VSe_2_ at such interfaces is significantly less reported in the literature compared to the well-documented case of MoSe_2_. Furthermore, the pronounced intermixing between the V_2_O_*x*_ and Se layers prevented conclusive SAED analysis and made it difficult to resolve distinct crystalline phases at the interface. Additionally, the inherently localized nature of TEM-based techniques also limits the detection of sparse or nonuniform interfacial selenide phases. Nevertheless, the spontaneous formation of TMD interlayers is frequently observed in high work function oxide systems, particularly at chalcogen-rich interfaces. This recurring behavior, combined with the strong intermixing observed at the V_2_O_*x*_/Se interface, suggests that a VSe_2_ or mixed selenide-oxide interlayer may have formed at room temperature, although conclusive evidence could not be obtained in this study.

To investigate the charge dynamics in the device, we tried to reconstruct the energy band diagrams of the two optimized architectures based on MoO_*x*_ and V_2_O_*x*_. For this purpose, the electronic work function and valence band maximum (VBM) of each layer of our devices were measured via UPS (Table S6). A bias voltage of −10 V was applied to the sample to decouple the measured work function from the spectrometer (Figure 6a). The WF was determined from the energy difference between the low-energy secondary electron cutoff (SECO) and the Fermi level, subtracted from the photon energy of the incident UV light. The VBM-E_F_ was obtained by fitting the HOMO edge slope in the region near the Fermi level (Figure 6b). Surface photovoltage effects and Fermi-level pinning were not taken into account in this analysis. The conduction band minimum (CBM) of each material was estimated by adding the optical band gap to the VBM position (Figure 6c). The band gap values were obtained by extrapolating Tauc plots derived from UV–Vis transmission spectra (Figure S7).

**Figure 6:**
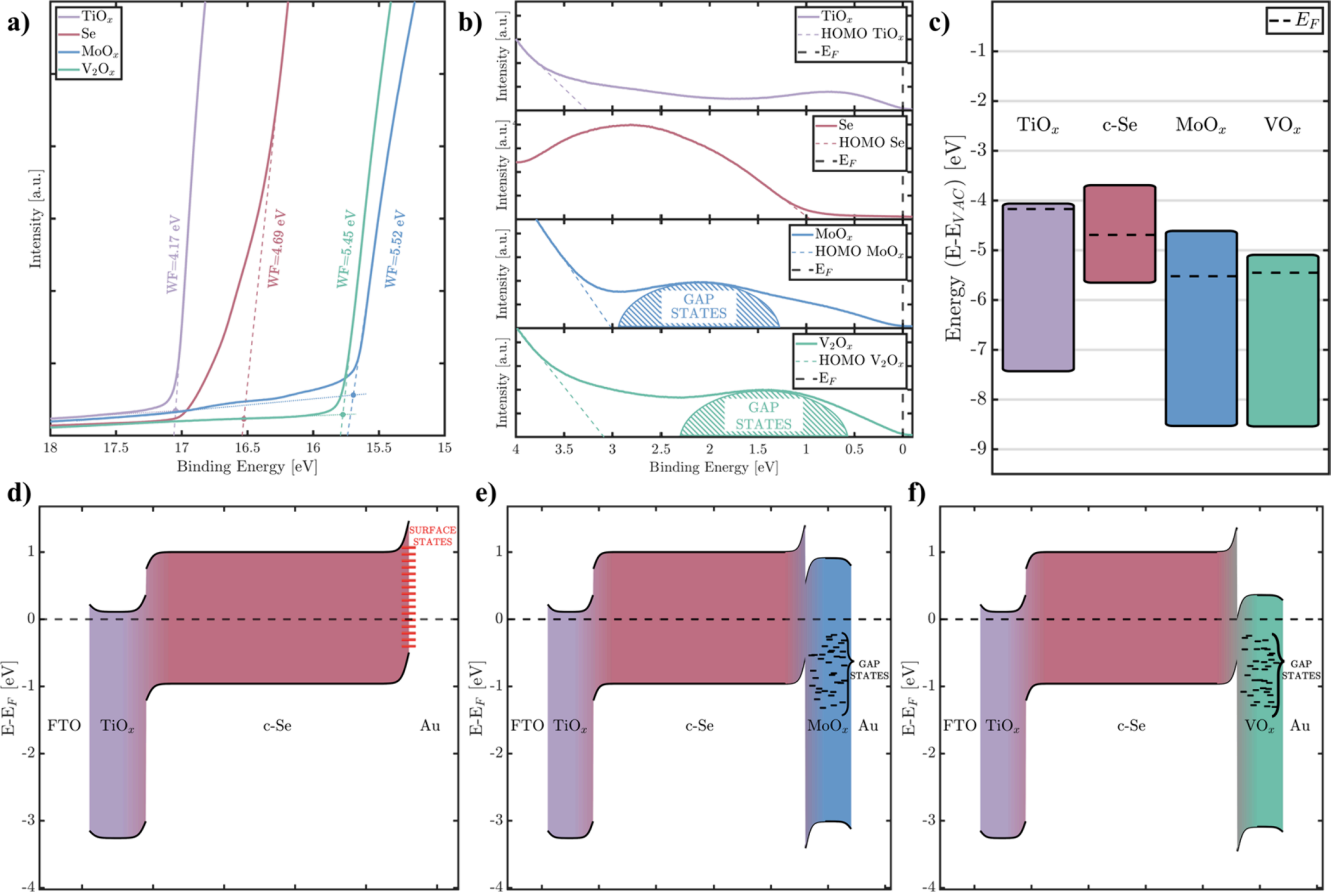
(a) SECO edge of the ultraviolet photoemission spectroscopy measurements with −10 V bias of TiO_2_, Se, MoO_*x*_, and V_2_O_*x*_ thin-films. (b) HOMO edge of the unbiased UPS measurements of the same layers. (c) Band structure of each of the layers obtained with the UPS and UV–vis measurements. Speculative band diagram of the (d) No HTL and optimized (e) MoO_*x*_ and (f) V_2_O_*x*_ devices.

Based on our analysis, we observe that the TiO_*x*_ layer deposited by spray pyrolysis is highly n-type, while the selenium absorber is essentially intrinsic under dark conditions as seen in the literature.^10^ This low doping under low illumination conditions is attributed to carrier freeze out that has been reported for Se absorbers.^50^ Both MoO_*x*_ and V_2_O_*x*_ are also n-type semiconductors; however, V_2_O_*x*_ exhibits a significantly higher doping concentration. Despite this difference, the WF and VBM of both TMOs are nearly identical. Additionally, the UPS spectra of both HTL oxides show a noticeable feature within the band gap region, attributed to intragap defect states, commonly associated with oxygen vacancies for these types of oxides.^51^

Using the extracted electronic parameters, we reconstructed the energy band diagrams of the two optimized devices (Figure 6 d–f) using Anderson’s rule.^52^ As shown, the resulting diagrams are quite similar, reflecting the electronic structures of MoO_*x*_ and V_2_O_*x*_. While Anderson’s model and classical drift-diffusion theory do not fully describe hole extraction through the TMO layers, they are useful for illustrating the formation of an electron-blocking barrier. This barrier results from the large work function of the oxides relative to that of selenium, which causes substantial upward band bending at the TMO/Se interface, thereby enabling hole selectivity. Hole extraction is believed to occur via defect-mediated hopping through intragap states.^32,43^ Given the large WF mismatch, the formation of an interfacial dipole between the absorber and high WF TMO is often assumed. This dipole reduces the effective built-in voltage of the junction and provides a more accurate representation of the interface, particularly when Anderson’s alignment suggests a nearly degenerate contact, as is nearly the case in the V_2_O_*x*_ band diagram.^26^ In addition, the possibility of forming an ultrathin TMD interlayer at the Se-TMO interface should be considered. Unlike studies in which deliberate thermal treatments induce extensive interfacial reactions, here the selenide layer remains much thinner: MoSe_2_ can only be detected as an ultrathin layer in HRTEM, and the presence of a comparable VSe_2_ layer appears to be similarly limited. Given their limited thickness, these interfacial selenides are more plausibly interpreted as passivating layers at the Se/TMO junction rather than as dominant contributors to the band alignment. Instead, the overall interface energetics are expected to be governed primarily by the oxide layers, owing to their greater thickness and large work function mismatch, which likely creates a large charge accumulation in the back, historically known as the back surface field (BSF). Furthermore, the spontaneous nature of selenide formation suggests that, provided the TMO layer is sufficiently thick (∼1–5 nm), an ultrathin interlayer will consistently emerge. Consequently, beyond a certain oxide thickness, this interfacial selenide is unlikely to strongly influence the optimum HTL thickness, although it may still play a beneficial role in local passivation and recombination suppression. This passivation effect is also reflected in the device metrics (Table 1), where cells incorporating TMO HTLs exhibit improved ideality factors together with enhanced apparent series and shunt resistance. This passivation effect may originate from the intrinsic 2D nature of TMDs, which effectively suppresses surface dangling bonds.^53–55^

In contrast, Figure 6d shows the band alignment of a reference device lacking an HTL. Although some upward band bending is still observed due to the high work function of the gold contact, the Se surface remains unpassivated. Consequently, this interface likely hosts a high density of defect states, which can result in Fermi level pinning and increased surface recombination, consistent with the Schottky-like behavior of the Au/Se junction.

## Conclusion

This study demonstrates that high work function transition metal oxides, specifically MoO_*x*_, WO_*x*_, and V_2_O_*x*_, serve as effective hole transport layers for selenium-based solar cells, providing substantial performance enhancements under both standard and indoor lighting conditions. Among the tested materials, MoO_*x*_ and V_2_O_*x*_ stood out, enabling power conversion efficiency of 5.5% and 5.3%, respectively, under AM1.5G illumination and exceeding 10% under typical indoor lighting. The improvements are primarily attributed to increased fill factors and open-circuit voltages, driven by enhanced shunt resistance and reduced series resistance.

Cross-sectional STEM-EDS and UPS analyses revealed strong interfacial interactions between selenium and the TMO layers. In particular, selected area electron diffraction (SAED) provided direct evidence of the spontaneous formation of MoSe_2_ at the MoO_*x*_/Se interface, even without substrate heating. While no conclusive SAED evidence was obtained for VSe_2_ in the V_2_O_*x*_-based devices, the extensive intermixing observed at the interface, along with the chemical similarity of the systems, strongly suggests that VSe_2_ may also form under similar conditions. These transition metal selenide interlayers likely contribute to improved hole extraction and suppressed interfacial recombination.

These results highlight the potential of inorganic HTLs to unlock new efficiencies in SeSCs and make them viable for emerging applications, such as indoor photovoltaics and tandem solar cells. By elucidating the physical and chemical interactions at the Se/TMO interface, this work provides a foundation for further device engineering and points to promising directions for the development of low-temperature, wide band gap photovoltaic technologies.

## Supplementary Material



## Notes

X-ray diffraction pattern of the crystallized selenium absorber, statistical analysis of photovoltaic parameters for HTL thickness optimization, single-diode circuital model, optoelectronic parameters for thickness optimization and EQE analysis, photovoltaic data used to construct indoor performance maps, *J*_SC_ indoor maps for different HTLs, work function and band structure parameters of device layers, STEM-EDX analysis of the 10 nm V_2_O_*x*_ device, and Tauc plot band gap fittings of all layers (PDF)

## References

[1] Smith W. (1873). Effect of
Light on Selenium during the Passage of an Electric Current. Nature.

[2] Adams W. G. (1877). The Action of Light
on Selenium. Proc. R. Soc.
London.

[3] Fritts C. E. (1883). On a New
Form of Selenium Cell, and Some Electrical Discoveries Made by Its
Use. Am. J. Sci..

[4] Green M. A. (2019). How Did
Solar Cells Get So Cheap?. Joule.

[5] Bhatnagar A. K., Reddy K. V., Srivastava V. (1985). Optical Energy
Gap of Amorphous Selenium:
Effect of Annealing. J. Phys. D Appl. Phys..

[6] Lu W., Li Z., Feng M., Yan H. J., Yan B., Hu L., Zhang X., Liu S., Hu J. S., Xue D. J. (2022). Melt- and
Air-Processed Selenium Thin-Film Solar Cells. Sci. China Chem..

[7] Green M. A., Dunlop E. D., Yoshita M., Kopidakis N., Bothe K., Siefer G., Hao X., Jiang J. Y. (2025). Solar Cell
Efficiency Tables (Version 66). Prog. Photovoltaics.

[8] Shockley W., Queisser H. J. (1961). Detailed Balance
Limit of Efficiency of Pn Junction
Solar Cells Additional Information. J. Appl.
Phys..

[9] Richter A., Hermle M., Glunz S. W. (2013). Reassessment
of the Limiting Efficiency
for Crystalline Silicon Solar Cells. IEEE J.
Photovolt.

[10] Nielsen R., Crovetto A., Assar A., Hansen O., Chorkendorff I., Vesborg P. C. K. (2024). Monolithic Selenium/Silicon Tandem
Solar Cells. PRX Energy.

[11] Tamin C., Chevalier C., Fave A. (2024). Preliminary Development of Selenium-Silicon
Tandem Solar Cells. La 14e édition des
Journées Nationales du PhotoVoltaïque.

[12] Mathuna C. O, O’Donnell T., Martinez-Catala R. V., Rohan J., O’Flynn B. (2008). Energy Scavenging
for Long-Term Deployable Wireless Sensor Networks. Talanta.

[13] Chakraborty A., Lucarelli G., Xu J., Skafi Z., Castro-Hermosa S., Kaveramma A. B., Balakrishna R. G., Brown T. M. (2024). Photovoltaics for
Indoor Energy Harvesting. Nano Energy.

[14] Müller M. F., Freunek M., Reindl L. M. (2013). Maximum
Efficiencies of Indoor Photovoltaic
Devices. IEEE J. Photovolt.

[15] Wei Z., Lu W., Li Z., Feng M., Yan B., Hu J. S., Xue D. J. (2023). Low-Cost
and High-Performance Selenium Indoor Photovoltaics. J. Mater. Chem. A Mater..

[16] Yan B., Liu X., Lu W., Feng M., Yan H.-J., Li Z., Liu S., Wang C., Hu J.-S., Xue D.-J. (2022). Indoor Photovoltaics
Awaken the World’s First Solar Cells. Sci. Adv..

[17] Nakada T., Kunioka A. (1985). Polycrystalline Thin-Film Tio2/Se Solar Cells. Jpn. J. Appl. Phys..

[18] Todorov T. K., Singh S., Bishop D. M., Gunawan O., Lee Y. S., Gershon T. S., Brew K. W., Antunez P. D., Haight R. (2017). Ultrathin
High Band Gap Solar Cells with Improved Efficiencies from the World’s
Oldest Photovoltaic Material. Nat. Commun..

[19] Lu W., Feng M., Li Z., Yan B., Wang S., Wen X., An X., Liu S., Hu J.-S., Xue D.-J. (2024). Ordering
One-Dimensional Chains Enables Efficient Selenium Photovoltaics. Joule.

[20] Liu Q., Wang X., Li Z., Lu W., Wen X., An X., Feng M., Yan H., Hu J., Xue D. (2025). Standing 1D
Chains Enable Efficient Wide-Bandgap Selenium Solar Cells. Adv. Mater..

[21] Lu W., Li Z., Feng M., Wei J., Wen X., An X., Wei Z., Lin Y., Hu J.-S., Xue D.-J. (2025). Lanthanide-Like
Contraction Enables the Fabrication of High-Purity Selenium Films
for Efficient Indoor Photovoltaics. Angew. Chem.-Int.
Ed..

[22] Caño I., Torrens A., Segura-Blanch O., Maggi E., Jiménez-Arguijo A., El Khouja O., Navarro-Güell A., Rovira D., Aroldi A., Asensi J., Alcobé X., Puigjaner C., Schwiddessen R., Schorr S., Pérez-Rodriguez A., Sylla D., Jehl Z., Saucedo E., Placidi M. (2025). Oriented for
Efficiency: Textured Se Thin Films Fabricated by Vapor Transport Deposition
for Emerging Photovoltaic Applications. Adv.
Energy Sustain. Res..

[23] Nielsen R., Hemmingsen T. H., Bonczyk T. G., Hansen O., Chorkendorff I., Vesborg P. C. K. (2023). Laser-Annealing and Solid-Phase Epitaxy of Selenium
Thin-Film Solar Cells. ACS Appl. Energy Mater..

[24] Youngman T. H., Nielsen R., Crovetto A., Seger B., Hansen O., Chorkendorff I., Vesborg P. C. K. (2021). Semitransparent Selenium Solar Cells
as a Top Cell for Tandem Photovoltaics. Sol.
RRL.

[25] Nielsen R. S., Schleuning M., Karalis O., Hemmingsen T. H., Hansen O., Chorkendorff I., Unold T., Vesborg P. C. K. (2024). Increasing
the Collection Efficiency in Selenium Thin-Film Solar Cells Using
a Closed-Space Annealing Strategy. ACS Appl.
Energy Mater..

[26] Gerling L. G., Mahato S., Morales-Vilches A., Masmitja G., Ortega P., Voz C., Alcubilla R., Puigdollers J. (2016). Transition Metal Oxides as Hole-Selective
Contacts in Silicon Heterojunctions Solar Cells. Sol. Energy Mater. Sol. Cells.

[27] Simchi H., McCandless B. E., Meng T., Shafarman W. N. (2014). Structure
and Interface Chemistry of MoO3 Back Contacts in Cu­(In,Ga)­Se2 Thin
Film Solar Cells. J. Appl. Phys..

[28] Hou F., Su Z., Jin F., Yan X., Wang L., Zhao H., Zhu J., Chu B., Li W. (2015). Efficient and Stable Planar Heterojunction
Perovskite Solar Cells with an MoO 3 /PEDOT:PSS Hole Transporting
Layer. Nanoscale.

[29] Schulz P., Tiepelt J. O., Christians J. A., Levine I., Edri E., Sanehira E. M., Hodes G., Cahen D., Kahn A. (2016). High-Work-Function
Molybdenum Oxide Hole Extraction Contacts in Hybrid Organic-Inorganic
Perovskite Solar Cells. ACS Appl. Mater. Interfaces.

[30] Wang Z., Wang Y., Taghipour N., Peng L., Konstantatos G. (2022). Ag-Refined
Kesterite in Superstrate Solar Cell Configuration with 9.7% Power
Conversion Efficiency. Adv. Funct Mater..

[31] Lin Y., Zhang Y., Magomedov A., Gkogkosi E., Zhang J., Zheng X., El-Labban A., Barlow S., Getautis V., Wang E., Tsetseris L., Marder S. R., Mcculloch I., Anthopoulos T. D. (2023). 18.73%
Efficient and Stable Inverted Organic Photovoltaics
Featuring a Hybrid Hole-Extraction Layer. Mater.
Horiz..

[32] Xing Y., Guo H., Liu J., Zhang S., Qiu J., Yuan N., Ding J. (2022). High-Efficiency
Sb 2 (S,Se) 3 Solar Cells with MoO 3 as a Hole-Transport
Layer. J. Alloy. Compd..

[33] Gong Y., Dong Y., Zhao B., Yu R., Hu S., Tan Z. (2020). Diverse Applications of MoO 3 for High Performance
Organic Photovoltaics:
Fundamentals, Processes and Optimization Strategies. J. Mater. Chem. A.

[34] Qiu L., Chen K., Yang D., Zhang M., Hao X., Li W., Zhang J., Wang W. (2021). Metal Copper Induced the Phase Transition
of MoO3 to MoO2 Thin Films for the CdTe Solar Cells. Mater. Sci. Semicond Process.

[35] Thomas L., Don C. H., Major J. D. (2022). An Investigation
into the Optimal
Device Design for Selenium Solar Cells. Energy
Rep..

[36] Liu W., Yu F., Fan W., Li W.-s., Zhang Q. (2021). Employing Equivalent
Circuit Models to Study the Performance of Selenium-Based Solar Cells
with Polymers as Hole Transport Layers. Small.

[37] Liu W., Yu F., Fan W., Zhang Q. (2020). Improved Stability and Efficiency
of Polymer-Based Selenium Solar Cells through the Usage of Tin­(Iv)
Oxide in the Electron Transport Layers and the Analysis of Aging Dynamics. Phys. Chem. Chem. Phys..

[38] Wang K., Shi Y., Zhang H., Xing Y., Dong Q., Ma T. (2014). Selenium as
a Photoabsorber for Inorganic-Organic Hybrid Solar Cells. Phys. Chem. Chem. Phys..

[39] Placidi M., Torrens A., Jehl Li-Kao Z., Lopez-Garcia A., Segura O., Gong Y., Jimenez-Arguijo A., Caño I., Giraldo S., Saucedo E., Alvarez G., Sanchez Y., Spalatu N., Oja I., Artegiani E., Romeo A., Scaffidi R., Perez-Rodriguez A. (2025). Benchmarking
Inorganic Thin-Film Photovoltaics Technologies for Indoor Applications. Sol. RRL.

[40] Gong Y., Jimenez-Arguijo A., Caño I., Scaffidi R., Malerba C., Valentini M., Payno D., Navarro-Güell A., Segura-Blanch O., Flandre D., Vermang B., Perez-Rodriguez A., Giraldo S., Placidi M., Jehl Li-Kao Z., Saucedo E. (2025). Attaining 15.1% Efficiency in Cu2ZnSnS4 Solar Cells
Under Indoor Conditions Through Sodium and Lithium Codoping. Sol. RRL.

[41] Li-Kao, Z. J. ; Tiwari, K. J. ; Giraldo, S. ; Placidi, M. ; Gong, Y. ; Basak, A. ; Kobayashi, T. ; Major, J. ; Saucedo, E. The Dilemma of Standardizing Indoor Photovoltaic Characterisation: Embracing Diversity for Powering the IoT. ArXiv 2024; 1

[42] Hoye R. L., Koutsourakis G., Freitag M., Jehl Li-Kao Z., Aliwell S., Bellanger M., Brown T. M., Brunetti F., Carnie M. J., Chakraborty A., Grancini G., Kärhä P., Kauer M., Kirchartz T., Lin C.-T., Lira-Cantú M., Long Y.-S., Raga S. R., Saucedo E., Vivo P., Woei Leow S., Wojciechowski K., Zampetti A., Zhou R., Züfle S., Burwell G. (2025). Reaching a Consensus on Indoor Photovoltaics
Testing. Joule.

[43] Gerling L. G., Voz C., Alcubilla R., Puigdollers J. (2017). Origin of Passivation in Hole-Selective
Transition Metal Oxides for Crystalline Silicon Heterojunction Solar
Cells. J. Mater. Res..

[44] Jimenez
Arguijo A., Kim M., Giraldo S., Güell A. N., Tiwari K., Khouja O., Gong Y., Kobayashi T., Jehl Li Kao Z. J. (2025). Guidelines for the Design of Efficient and Injection-Resilient
Thin Film Photovoltaic Converters for Indoor Applications. J. Phys-Energy.

[45] Theelen M., Broos R. J. P., Hovestad A. (2022). The Influence of Atmospheric Species
on the Degradation of the Mo/MoSe2 Back Contact in CIGS Solar Cells. Mater. Chem. Phys..

[46] Duchatelet A., Savidand G., Vannier R. N., Lincot D. (2013). Optimization of MoSe2
Formation for Cu­(In,Ga)Se 2-Based Solar Cells by Using Thin Superficial
Molybdenum Oxide Barrier Layers. Thin Solid
Films.

[47] Li J., Huang J., Ma F., Sun H., Cong J., Privat K., Webster R. F., Cheong S., Yao Y., Chin R. L., Yuan X., He M., Sun K., Li H., Mai Y., Hameiri Z., Ekins-Daukes N. J., Tilley R. D., Unold T., Green M. A., Hao X. (2022). Unveiling
Microscopic Carrier Loss Mechanisms in 12% Efficient Cu2ZnSnSe4 Solar
Cells. Nat. Energy.

[48] Ramírez-Velasco S., González-Castillo J. R., Ayala-Mató F., Hernández-Calderón V., Jiménez-Olarte D., Vigil-Galán O. (2022). Back Contact
Modification in Sb2Se3 Solar Cells: The
Effect of a Thin Layer of MoSe2. Thin Solid
Films.

[49] Bao F., Liu L., Wang X., Xiao B., Li H., Yang H., Shen K., Mai Y. (2025). Modification of the Se/MoOx Rear
Interface for Efficient Wide-Band-Gap Trigonal Selenium Solar Cells. ACS Appl. Mater. Interfaces.

[50] Nielsen R. S., Gunawan O., Todorov T., Møller C. B., Hansen O., Vesborg P. C. K. (2025). Variable-Temperature and Carrier-Resolved
Photo-Hall Measurements of High-Performance Selenium Thin-Film Solar
Cells. Phys. Rev. B.

[51] Greiner M. T., Chai L., Helander M. G., Tang W. M., Lu Z. H. (2012). Transition
Metal Oxide Work Functions: The Influence of Cation Oxidation State
and Oxygen Vacancies. Adv. Funct Mater..

[52] Anderson R. L. (1960). Germanium-Gallium
Arsenide Heterojunctions [Letter to the Editor]. IBM J. Res. Dev.

[53] Wang Y., Wang R., Wang G., Fu G., Zheng L., Song Q., Zhou Y., Pan J., Jiang M., Peng S. (2025). 2D MoSe2 Capping Layer Passivation for High-Efficiency Large-Area
CdTe Solar Cells. J. Power Sources.

[54] Mondal S., Eguchi N., Nishimura N., Hinuma Y., Yamamoto K., Kogo A., Murakami T. N., Kanda H. (2024). Mixed 2D-Cation Passivation
towards Improved Durability of Perovskite Solar Cells and Dynamics
of 2D-Perovskites under Light Irradiation and at High Temperature. Sustain Energ Fuels.

[55] Shirzadi E., Ansari F., Jinno H., Tian S., Ouellette O., Eickemeyer F. T., Carlsen B., Van Muyden A., Kanda H., Shibayama N., Tirani F. F., Grätzel M., Hagfeldt A., Nazeeruddin M. K., Dyson P. J. (2023). High-Work-Function
2D Perovskites as Passivation Agents in Perovskite Solar Cells. ACS Energy Lett..

