# Supplementary material for: Understanding the Role of Transition Metal Oxides as Hole-Selective Contacts for Enhanced Efficiency in Selenium Solar Cells
Source: ACS Appl Energy Mater. 2025 Nov 6;8(22):16729–38. doi: 10.1021/acsaem.5c02637 (PMC12648467; doi:10.1021/acsaem.5c02637)
Supplement: Supplementary file 1 [file ae5c02637_si_001.pdf]

Supporting Information: Understanding the Role of Transition Metal Oxides as Hole-Selective Contacts for enhanced efficiency in Selenium Solar Cells

Oriol Segura Blanch*[1,2], Arnau Torrens[1,2], Ivan Caño Prades[1,2], Alex Jimenez-Arguijo[1,2], Laura Garcia-Carreras[3,4] Lorenzo Calvo-Barrio[3,4], José Miguel Asensi[5], Joaquim Puigdollers[1], Marcel Placidi[1,2,6], Edgardo Saucedo[1,2]

1. *Universitat Politècnica de Catalunya (UPC), Photovoltaic Lab – Micro and Nano Technologies Group (MNT), Electronic Engineering Department, EEBE, Av Eduard Maristany 10-14, Barcelona 08019, Catalonia, Spain*
2. *Universitat Politècnica de Catalunya (UPC), Barcelona Center in Multiscale Science & Engineering, Av Eduard Maristany 10-14, Barcelona 08019, Catalonia, Spain*
3. *Centres Científics i Tecnològics(CCiTUB), Universitat de Barcelona, Carrer de Lluís Solé i Sabarís 1, 08028 Barcelona,Spain;*
4. *IN2UB, Departament d'Enginyeria Electrònica i Biomèdica, Universitat de Barcelona, Carrer de Martí i Franquès 1, 08028 Barcelona, Spain*
5. *IN2UB,Departament de Física AplicadaUniversitat de Barcelona, Carrer de Martí i Franquès 1, 08028 Barcelona, Spain*
6. *Institut de Recerca en Energia de Catalunya (IREC), Jardins de les Dones de Negre 1, Sant Adrià del Besòs 08930,Catalonia, Spain*

***Corresponding autor:** Oriol Segura-Blanch, oriol.segura.blanch@upc.edu

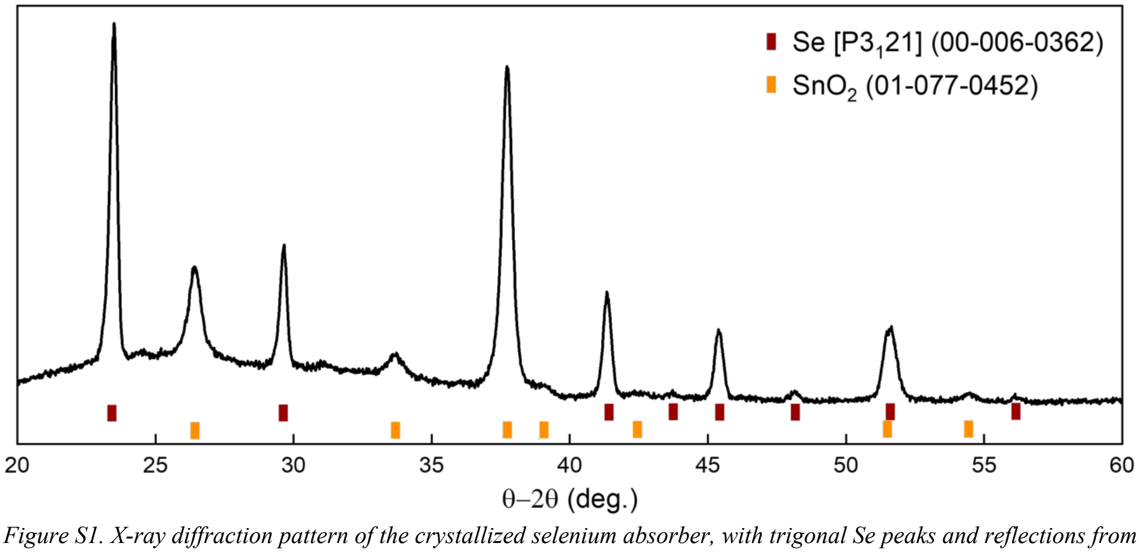

Figure S1. X-ray diffraction pattern of the crystallized selenium absorber, with trigonal Se peaks and reflections from the FTO substrate. Measurements were performed using a Bruker D8 Advance diffractometer in the 20–60° range, with a step size of 0.02° and an acquisition time of 1 s per point.

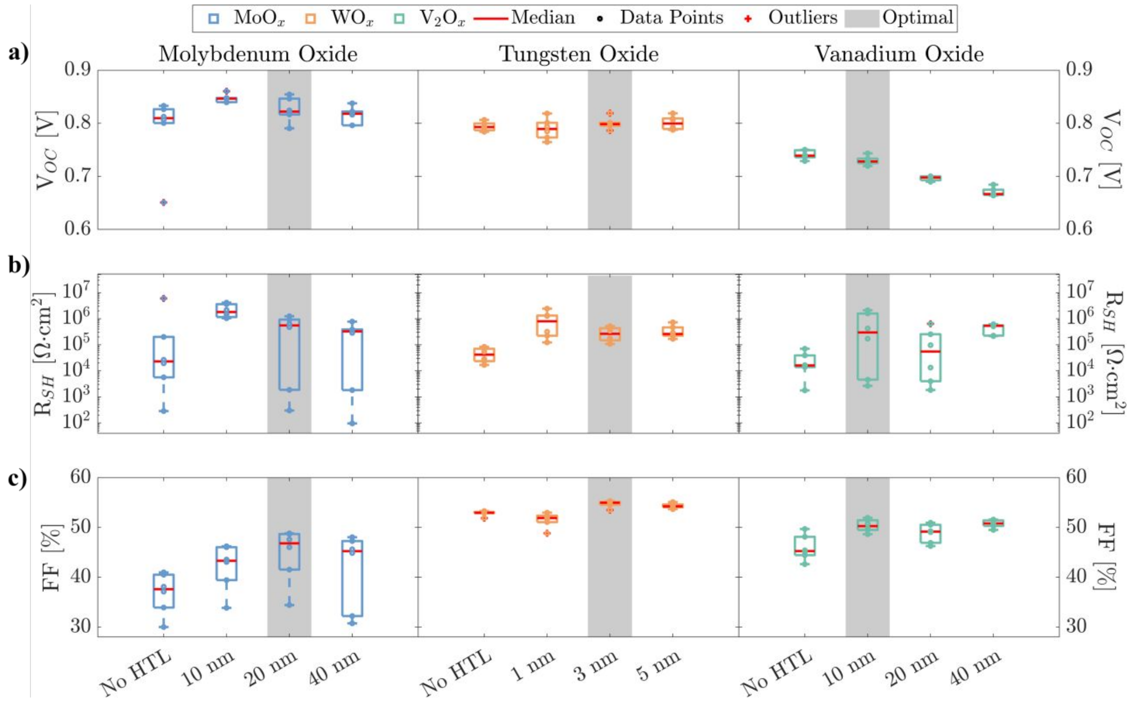

Figure S2. Statistics of the a) V_{OC}, b)R_{SH}, and c) FF of the thickness optimization of the MoO_x, WO_x and VO_x hole transport layers for the Pre-crystalized samples.

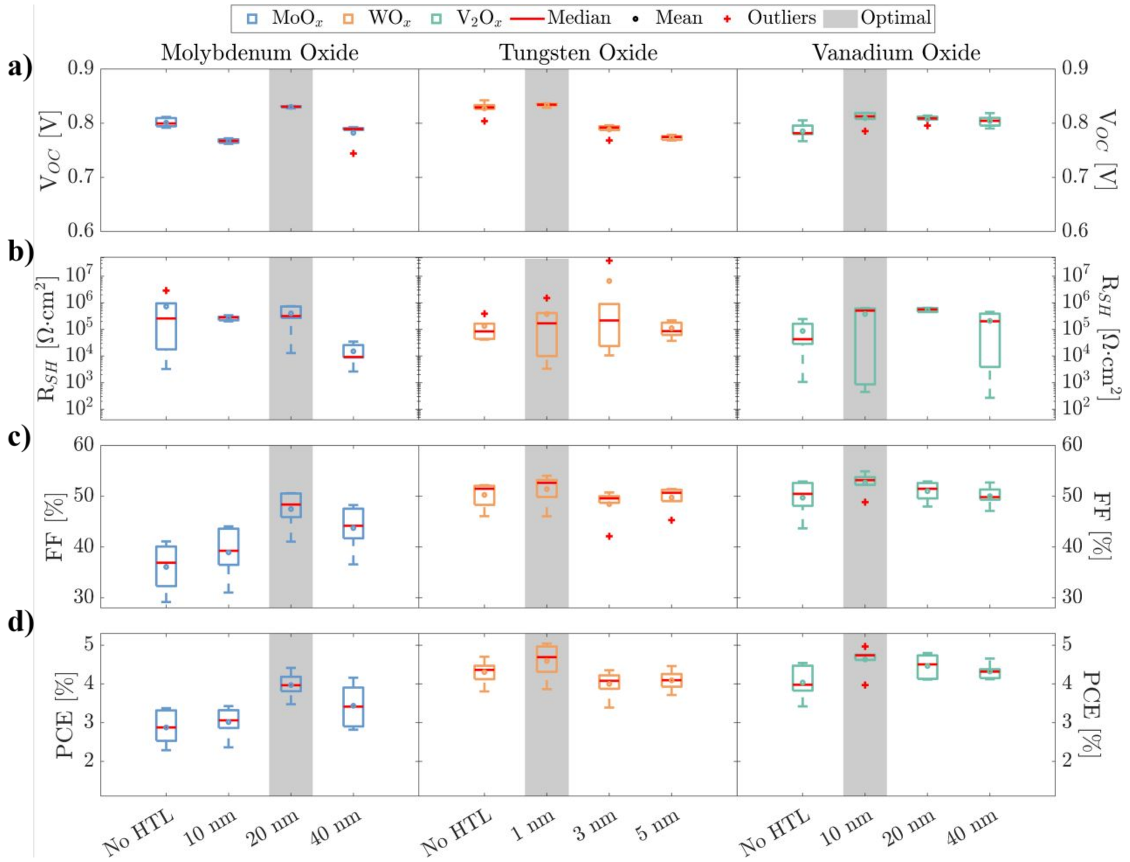

Figure S3. Statistics of the a) V_{OC}, b)R_{SH}, c) FF and d) PCE of the thickness optimization of the MoO_x, WO_x and VO_x hole transport layers for the Post-crystalized samples.

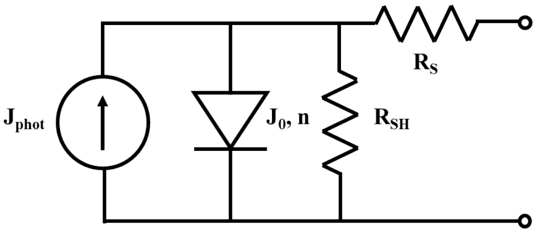

Figure S4. Single diode circuital of a solar cell consisting on a current generator (J_{phot}), a non-ideal diode (J_0, n), shunt resistance (R_{SH}) and series resistance (R_S).

Table S1. Optoelectronic parameters for the HTL thickness optimization of MoO_x, WO_x, and VO_x. The optimal thickness in each case is shaded in gray. Values represent the mean over six different cells and the error corresponds to the standard deviation (except for the error of the J_0 and R_{SH} where the standard error of the mean is used due to their logarithmic nature).

	HTL	Thickness	V_{OC} [mV]	J_{SC} [mA/cm²]	FF [%]	PCE [%]	R_S [Ω·cm²]	R_{SH} [MΩ·cm²]
Pre-Crystalized	MoO_x	No HTL	790 ± 70	10.3 ± 0.3	36.8 ± 4.2	3.0 ± 0.5	62.3 ± 29.3	1.05 ± 1.00
		10 nm	850 ± 10	10.7 ± 0.5	42.0 ± 4.7	3.8 ± 0.5	63.0 ± 33.0	2.26 ± 0.54
		20 nm	830 ± 20	10.9 ± 0.3	44.5 ± 5.6	4.0 ± 0.5	41.4 ± 24.5	0.54 ± 0.20
		40 nm	750 ± 170	10.7 ± 0.7	41.5 ± 7.8	3.5 ± 1.3	42.9 ± 26.9	0.30 ± 0.12
	WO_x	No HTL	790 ± 10	10.2 ± 0.3	52.8 ± 0.5	4.3 ± 0.1	29.3 ± 3.8	0.046 ± 0.011
		1 nm	790 ± 20	10.5 ± 0.4	51.5 ± 1.4	4.3 ± 0.2	64. 6 ± 22.2	0.95 ± 0.37
		3 nm	800 ± 10	10.5 ± 0.4	54.7 ± 0.7	4.6 ± 0.2	19.1 ± 6.8	0. 29 ± 0.07
		5 nm	800 ± 10	10.5 ± 0.3	54.3 ± 0.5	4.6 ± 0.2	24.0 ± 3.6	0.35 ± 0.09
	V_2O_x	No HTL	740 ± 10	9.5 ± 0.2	45.9 ± 2.6	3.2 ± 0.2	20.6 ± 4.1	0.026 ± 0.010
		10 nm	730 ± 10	10.2 ± 0.2	50.3 ± 1.3	3.7 ± 0.2	9.8 ± 1.7	0.72 ± 0.37
		20 nm	700 ± 5	10.8 ± 0.2	48.8 ± 1.9	3.7 ± 0.2	8.2 ± 1.0	0.17 ± 0.10
		40 nm	670 ± 10	10.5 ± 0.4	50.7 ± 0.8	3.6 ± 0.2	5.8 ± 0.5	0.45 ± 0.07
Post-Crystalized	MoO_x	No HTL	800 ± 10	9.9 ± 0.4	36.1 ± 4.7	2.9 ± 0.4	78.2 ± 47.4	0.73 ± 0.46
		10 nm	770 ± 5	10.1 ± 0.2	39.0 ± 4.9	3.0 ± 0.4	47.2 ± 27.2	0.27 ± 0.02
		20 nm	830 ± 5	10.1 ± 0.4	47.5 ± 3.6	4.0 ± 0.3	37.6 ± 13.8	0.40 ± 0.12
		40 nm	780 ± 20	10.0 ± 0.6	43.7 ± 4.4	3.4 ± 0.5	35.7 ± 29.7	0.015 ± 0.005
	WO_x	No HTL	830 ± 10	10.3 ± 0.4	50.3 ± 2.5	4.3 ± 0.3	51.1 ± 28.5	0.13 ± 0.05
		1 nm	830 ± 5	10.7 ± 0.5	51.4 ± 3.0	4.6 ± 0.5	43.1 ± 16.5	0.38 ± 0.24
		3 nm	790 ± 5	10.5 ± 0.3	48.4 ± 3.2	4.0 ± 0.3	266.5 ± 25.1	6.59 ± 6.32
		5 nm	770 ± 5	10.6 ± 0.5	49.7 ± 2.3	4.1 ± 0.3	351.3 ± 150.7	0.11 ± 0.03
	V_2O_x	No HTL	790 ± 10	10.3 ± 0.5	49.7 ± 3.6	4.0 ± 0.4	25.6 ± 13.2	0.087 ± 0.039
		10 nm	810 ± 10	10.8 ± 0.3	52.7 ± 2.1	4.6 ± 0.3	12.4 ± 1.0	0.38 ± 0.12
		20 nm	810 ± 10	10.8 ± 0.4	51.0 ± 2.0	4.5 ± 0.3	17.0 ± 2.6	0.55 ± 0.04
		40 nm	800 ± 10	10.7 ± 0.2	50.0 ± 1.9	4.3 ± 0.2	19.2 ± 3.3	0.21 ± 0.01

Table S2 Optoeletronic parameters of the EQE spectras of the optimized cells for the 3 HTL configurations tested and the reference No HTL device for the Pre-crystalized samples.

HTL	J_{SC} [mA/cm²]	E_G [eV]	E_U [mV]
No HTL	9.82	1.98	34.45
MoO_x 20 nm	10.14	1.97	29.91
WO_x 3 nm	10.17	1.97	33.65
V_2O_x 10 nm	10.40	1.94	31.26

Table S3 Photovoltaic parameters used to construct the indoor performance maps of the No HTL sample.

Color Temperature	Incident Power [μW/cm²]	V_{OC} [mV]	J_{SC} [μA/cm²]	FF [%]	PCE [%]
2700 K	3119.66	607.2	696.8	55.97	7.59
	2142.09	586.2	498.1	56.09	7.65
	1287.39	557.5	309.6	55.29	7.41
	537.39	498.6	129.7	51.65	6.22
	88.68	333.6	21.5	41.13	3.33
3000 K	2905.98	604.6	640.2	56.27	7.50
	1936.97	583.3	448.4	55.35	7.48
	1141.03	553.8	275.1	55.34	7.39
	463.68	491.2	112.1	51.11	6.07
	58.01	283.7	14.2	36.61	2.53
4000 K	2852.56	609.3	657.4	56.03	7.87
	1903.85	585.9	457.1	56.22	7.91
	1115.38	554.3	272.4	54.83	7.42
	432.69	484.3	104.7	50.42	5.91
	48.08	265.8	11.7	35.72	2.31
5000 K	2884.62	613.2	675.7	56.03	8.05
	1952.99	589.1	471.7	55.93	7.96
	1141.03	554.3	280.6	54.26	7.40
	423.08	481.7	102.2	50.29	5.85
	48.08	256.0	11.7	36.23	2.26
6000 K	2628.21	603.6	602.1	56.43	7.80
	1733.97	582.4	420.9	55.60	7.86
	990.38	548.7	246.4	54.33	7.42
	330.13	462.8	80.1	48.45	5.44
	8.76	84.1	2.1	26.87	0.55

Table S4 Photovoltaic parameters used to construct the indoor performance maps of the 20 nm MoO_x sample.

Color Temperature	Incident Power [μW/cm²]	V_{OC} [mV]	J_{SC} [μA/cm²]	FF [%]	PCE [%]
2700 K	3119.66	623.2	682.2	57.58	7.85
	2142.09	602.4	489.1	57.38	7.89
	1287.39	570.9	304.3	57.05	7.70
	537.39	505.4	127.1	57.12	6.83
	88.68	382.5	21.0	56.79	5.13
3000 K	2905.98	623.2	629.3	57.97	7.82
	1936.97	603.3	441.4	57.88	7.89
	1141.03	566.2	270.8	57.52	7.71
	463.68	501.6	110.0	57.39	6.79
	58.01	361.1	13.7	56.42	4.79
4000 K	2852.56	629.1	643.6	58.09	8.25
	1903.85	603.9	448.0	57.88	8.23
	1115.38	567.7	267.4	57.52	7.83
	432.69	496.5	102.6	57.39	6.76
	48.08	351.1	11.3	56.	4.66
5000 K	2884.62	633.1	659.8	58.21	8.43
	1952.99	608.9	460.8	57.89	8.32
	1141.03	571.8	274.5	57.57	7.92
	423.08	496.6	100.1	57.44	6.75
	48.08	352.4	11.3	56.56	4.69
6000 K	2628.21	625.0	587.0	58.39	8.15
	1733.97	601.5	410.1	57.97	8.25
	990.38	563.3	240.3	57.65	7.88
	330.13	481.4	78.3	57.37	6.55
	8.76	262.3	2.0	52.56	3.18

Table S5 Photovoltaic parameters used to construct the indoor performance maps of the 10 nm V_2O_x sample.

Color Temperature	Incident Power [µW/cm^2]	V_{OC} [mV]	J_{SC} [µA/cm^2]	FF [%]	PCE [%]
2700 K	3055.56	726.6	671.3	59.70	9.53
	1245.73	698.8	304.9	59.27	10.14
	517.09	661.6	132.3	57.63	9.75
	83.65	565.1	24.6	51.16	8.51
3000 K	2841.88	730.4	619.4	59.96	9.54
	1101.50	698.0	272.1	59.13	10.20
	517.09	666.15	115.2	56.93	9.72
	83.65	540.1	16.7	49.45	8.22
4000 K	2777.78	734.1	635.4	60.14	10.10
	1075.85	698.5	269.9	59.21	10.38
	416.67	656.5	108.0	56.69	9.69
	44.44	527.4	14.0	48.83	8.14
5000 K	2799.15	735.9	652.3	60.20	10.32
	1100.43	700.2	277.5	59.33	10.48
	405.98	658.4	105.6	56.71	9.72
	44.66	527.4	14.1	48.98	8.15
6000 K	2534.19	731.1	584.0	60.36	10.17
	955.13	697.9	245.3	59.02	10.59
	317.31	648.5	84.0	55.84	9.59
	6.67	382.9	2.9	44.21	7.46

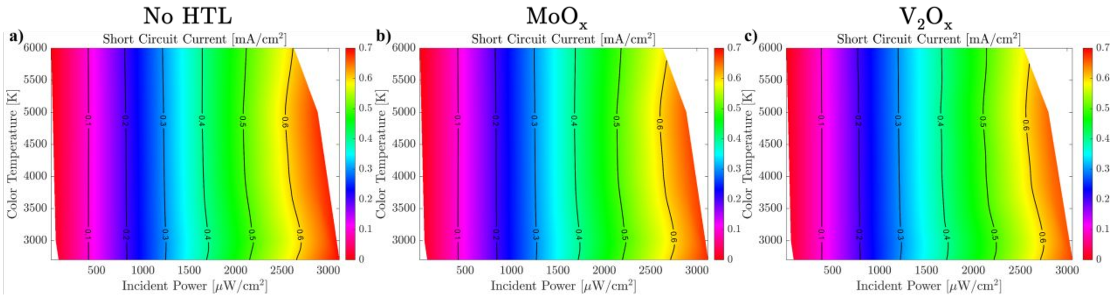

Figure S5. J_{SC} maps in indoor conditions of the a) No HTL, b) 20 nm MoO_x and c) 10 nm V_2O_x.

Table S6. Work function, valence band maximum relative to the Fermi level, and optical band gap of the different device layers.

	**WF [eV]**	**VBM-E_F [eV]**	**E_G [eV]**
TiO_x	4.17	3.26	3.35
Se	4.69	0.96	1.93
MoO_x	5.52	3.01	3.92
V_2O_x	5.45	3.09	3.45

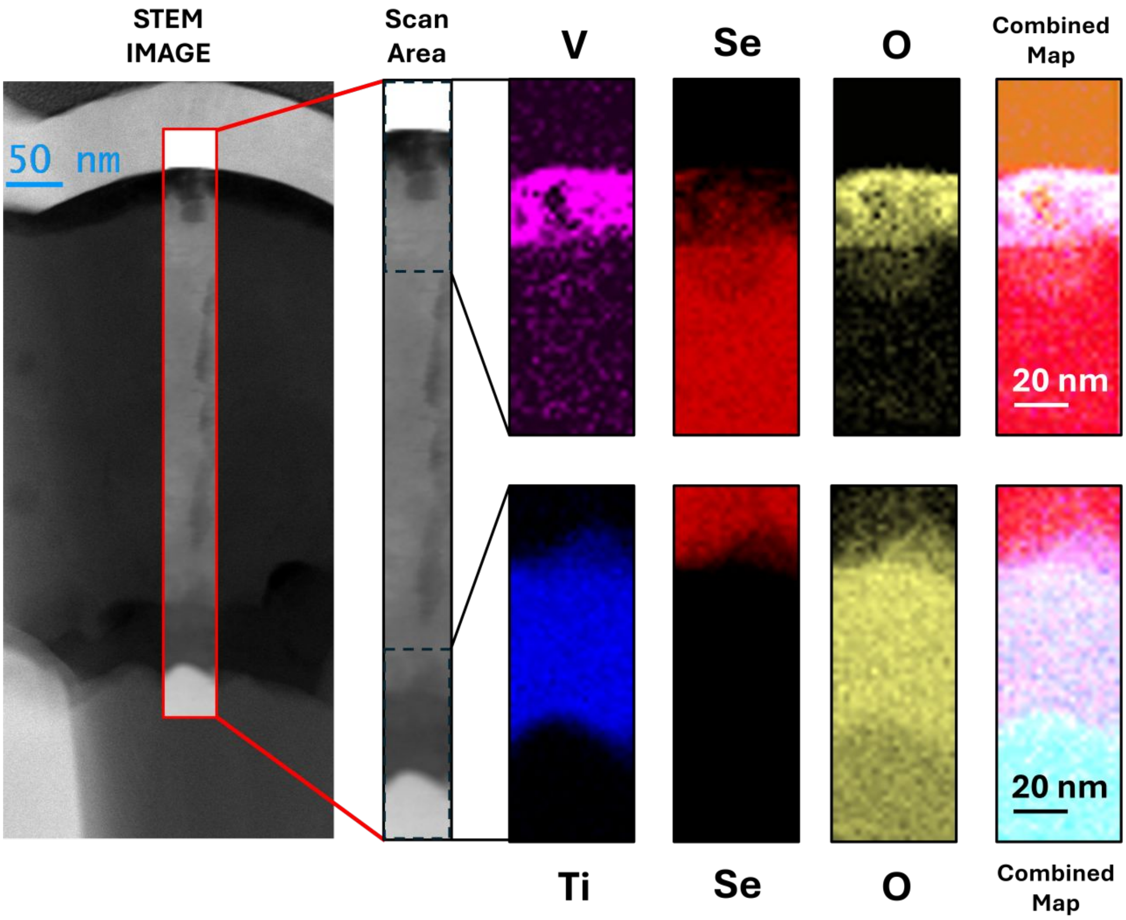

Figure S6. STEM image and EDX maps of the 10 nm V_2O_x device.

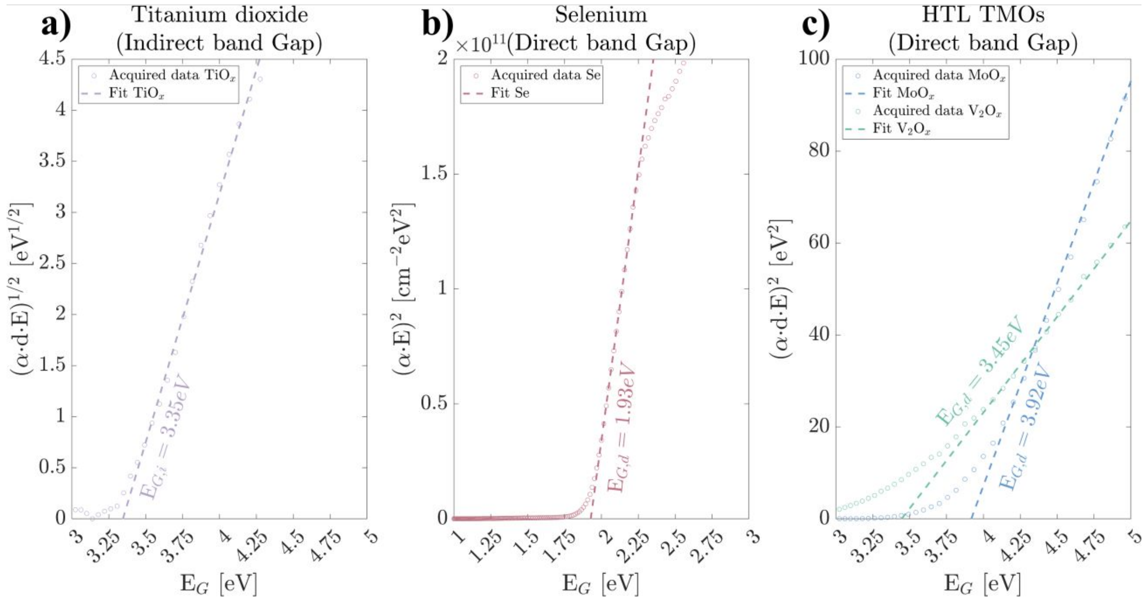

Figure S7.Band gap fitting and Tauc plots of a) TiO_x, b) Se, c) MoO_x and V_2O_x.
